# Anti-plasminogen antibodies in ANCA-associated vasculitis: An optimized anti-plasminogen assay

**DOI:** 10.1371/journal.pone.0207064

**Published:** 2018-11-12

**Authors:** Arda Göçeroğlu, Elsa Grenmyr, Annelies E. Berden, E. Christiaan Hagen, Donna Bunch, Yngve Sommarin, Jan A. Bruijn, Ingeborg M. Bajema, Jörgen Wieslander

**Affiliations:** 1 Department of Pathology, Leiden University Medical Center, Leiden, The Netherlands; 2 Euro Diagnostica, Malmö, Sweden; 3 Department of Nephrology, Meander Medical Center, Amersfoort, The Netherlands; 4 Kidney Center, University of North-Carolina, Chapel Hill, North Carolina, United States of America; Duke University School of Medicine, UNITED STATES

## Abstract

Anti-plasminogen antibodies (α-PLG) were previously detected in a subpopulation of anti-neutrophil cytoplasmic antibody (ANCA)-associated vasculitis (AAV) patients, showing a relation to renal lesions and outcome. Several studies showed different proportions of α-PLG positive AAV patients, possibly due to differences in the assays used. We here present a new, optimized α-PLG Enzyme-Linked Immuno Sorbent Assay (ELISA) and validate the presence of α-PLG in AAV. Different ELISA set-ups were tested regarding plasminogen (PLG) antigen, concentrations, coating buffers, blocking agents, and environmental conditions. Purified lysine-PLG (lys-PLG) showed better differentiation between positive samples and negative samples than glutamic acid-PLG (glu-PLG). Therefore, lys-PLG was used as coating antigen. With the optimized α-PLG ELISA we found α-PLG in 14.3% of the myeloperoxidase (MPO)-ANCA patients, whereas all our proteinase-3 (PR3)-ANCA patients tested in our new assay were negative. Concluding, in this study we have combined important technical findings and methods from previous studies to optimize the α-PLG assay, which can be used for future research purposes and will aid in uniform reporting of α-PLG status of patients.

## Introduction

Recently, the presence of anti-plasminogen antibodies (α-PLG) in patients with anti-neutrophil cytoplasmic antibody (ANCA)-associated vasculitis (AAV) received much attention, especially in relation to the nature and severity of renal lesions.[1–3] These antibodies inhibit fibrinolysis by disturbing the conversion of plasminogen (PLG) to plasmin.[1,2] A study on patients with AAV showed that patients with α-PLG had significantly more glomerular fibrinoid necrosis accompanied by worse renal function.[2] Evidently, the presence of α-PLG in AAV may be an important hallmark for a specific phenotype of the disease.[2,3] Three important studies on α-PLG in AAV reported differences in the proportion of α-PLG positive AAV patients ranging between 22%-43% for proteinase-3 (PR3)-AAV and 6%-27% for myeloperoxidase (MPO)-AAV.[1–3] It is possible that differences in α-PLG assays were to some extent responsible for these discrepant results. We therefore optimized the method for α-PLG Enzyme-Linked Immuno Sorbent Assay (ELISA) and with this new assay, we validated the presence of α-PLG in AAV.

## Materials and methods

### Positive controls

Eleven positive controls were derived from the studies of Bautz *et al*. and Berden *et al*.[1,2] These positive samples consisted of serum or plasma exchange (PEX) fluid. These patients had the following ANCA-specificities: 5 MPO-ANCA, 5 PR3-ANCA and 1 ANCA negative. These were detected with WIESLAB MPO-ANCA / MPO IU, WIESLAB Capture MPO-ANCA / CAP MPO IU, WIESLAB PR3-ANCA / PR3 IU and WIESLAB Capture PR3-ANCA / CAP PR3 IU (Euro Diagnostica, Malmö, Sweden). Patients had been diagnosed with AAV according to the 2012 revised International Chapel Hill Consensus Conference Nomenclature of Vasculitides.[4]

### Healthy and disease controls

Samples from 220 healthy controls were used during the different steps for optimizing the assay. Samples of 157 disease controls were used. Of these samples 77 were anti-beta-2 glycoprotein 1 (β2GP1) positive, which is an autoantibody found in systemic lupus erythematosus and anti-phospholipid syndrome.[5] The remaining 80 samples were positive for anti-cyclic citrullinated peptides (CCP), which is an autoantibody found in rheumatoid arthritis.[6] Samples from healthy and disease controls were collected at Euro Diagnostica, Malmö, Sweden.

### ANCA samples

For setting-up and optimizing the α-PLG assay 104 randomly selected samples of patients with ANCA positivity were used. Samples were not selected with respect to disease state. Of these samples 55 were PR3-ANCA positive and 49 were MPO-ANCA positive. These samples were collected at Euro Diagnostica, Malmö, Sweden. ANCA specificity of each patient was determined using WIESLAB MPO-ANCA / MPO IU, WIESLAB Capture MPO-ANCA / CAP MPO IU, WIESLAB PR3-ANCA / PR3 IU and WIESLAB Capture PR3-ANCA / CAP PR3 IU (Euro Diagnostica, Malmö, Sweden). The use of the samples in this study was approved by the Lund University ethics committee. All patients gave written informed consent to store samples for future development of analytical methods for the purpose of hospital care and treatment or similar activity. This study was conducted in accordance with the 1964 Declaration of Helsinki and subsequent amendments. This study was also performed according to the 'Netherlands Code of Conduct for Scientific Practice', an ethical code for performing observational studies with patient material approved by the Federatie van Medisch Wetenschappelijke Verenigingen (Federation of Medical Scientific Organisations) together with the legal and ethical committee of the Koninklijke Nederlandse Akademie van Wetenschappen (Royal Dutch Academy of Science) and the Nederlandse Organisatie voor Wetenschappelijk Onderzoek (Dutch Organisation for Scientific Research). The data of the patients were analyzed anonymously.

### Anti-plasminogen antibody assay

For developing an α-PLG assay we optimized each step in the assay by testing different alternatives for each step. These steps and their alternatives were:

- Coating material: glutamic acid plasminogen (glu-PLG), purified glu-PLG, lysine-plasminogen (lys-PLG) or purified lys-PLG obtained from Calbiochem and from Haematologic Technologies. Contaminating Immunoglobulin G (IgG) was removed from commercially obtained PLG by affinity chromatography on a Protein G HP SpinTrap column from GE Technologies using as binding buffer 0.01 phosphate, 0.15 M NaCl, 0.01 M EDTA pH 7.0.
- Coating buffer: 0,05 M Sodium Carbonate pH 9.5 or PBS.
- Blocking buffer: PBS with BSA 1%, gelatin 1% or Stabilcoat (Surmodics).
- Diluent: PBS with 0,05% Tween 20 and 2g/l BSA. Different serum dilutions (1:50, 1:100, 1:200, 1:400) and purified IgG from the serum samples were tested as primary antibodies.
- Wash: 0,15M NaCl with 0,05% Tween 20.
- Conjugated secondary antibodies: Sigma goat anti-human IgG HRP, Sigma goat anti-human IgG AP or Dako rabbit anti-human IgG HRP.
- Conjugate buffer: PBS with 0,05% Tween 20, 2g/l BSA and 1g/l bovine IgG.
- Substrate: para-Nitrophenylphosphate

We tested different suppliers of antigen, different temperatures (room temperature and 37°C) and different time frames (30, 60 and 120 minutes) for each step. Box 1 describes the optimized assay.

**Box 1. Anti-plasminogen antibody ELISA assay**

High binding microtiter plates were coated overnight with 3 μg/ml protein G purified lysine-plasminogen at room temperature. Carbonate buffer pH 9,6 was used as coating buffer. Plates were blocked for 60 minutes with 1% BSA in PBS at room temperature. After washing with PBS-Tween, serum and PEX fluid at 1/100 dilution were added in duplicates into appropriate microtiter wells and incubated at 37°C for 60 minutes. Wells were washed and incubated (30 minutes at 37°C) with a 1:5000 dilution of Sigma goat anti-human IgG-alkaline phosphatase. After washing, 4-nitrophenyl phosphate was used as the substrate at room temperature, and the 96-well microtiter plate was analyzed spectrophotometrically at 405 nm after 30 minutes.

### IgG purification from serum or PEX fluid

IgG was purified from the sera or PEX fluid using the Melon Gel IgG Purification Kit (Pierce Protein Research Products; Thermo Scientific). SDS-PAGE was used to confirm the integrity of isolated IgG and Coomassie blue staining was used to visualize isolated IgG.

### Testing different anti-human IgG conjugates

We tested different conjugates in varying concentrations to obtain the best signal of bound α-PLG on the plate coated with PLG. The conjugates tested were: Sigma goat anti-human IgG HRP 1/10000 and 1/20000, Sigma goat anti-human IgG AP 1/5000 and 1/10000, Dako rabbit anti-human IgG HRP 1/30000 and 1/50000.

### Glu-plasminogen inhibition assays

Two inhibition assays were performed:

1. Glu-PLG 2 μg/ml was coated and incubated with strong positive control serum 1/200, healthy control serum 1/200 or Sigma goat anti-human IgG AP 1/10000. The sera was mixed with an increasing amount of soluble PLG (0–10 μg/ml) and the conjugate was mixed with an increasing amount of human IgG (0–10 μg/ml) for inhibition.
2. Soluble glu-PLG for inhibition (0–10 μg/ml) was pre-incubated overnight with polyclonal rabbit antibodies (α-PLG and anti-Chromogranin-A (CgA)), human sera (strong positive control and healthy control) or diluent. This was then added to a plate coated with glu-PLG 1 μg/ml. Swein anti-rabbit AP and goat anti-human AP were used for signal detection.

### SDS-PAGE and Western blot

SDS-PAGE was performed with four proteins: unreduced glu-PLG, unreduced lys-PLG, reduced glu-PLG, reduced lys-PLG. Western blot was performed using rabbit α-PLG as specific antibody.

### Titration curves plasminogen coating

Plates were coated with glu-PLG, purified glu-PLG, lys-PLG and purified lys-PLG in concentrations varying from 0 to 5 μg/ml. Samples from four positive controls and four negative controls in dilutions of 1/100 and 1/200 were tested. In addition, rabbit α-PLG, rabbit anti-CgA and diluent were tested. We performed the titrations with and without BSA block.

### Statistical analysis

χ2-test was performed to compare the spectrophotometrical results of purfied glu-PLG and purified lys-PLG as coating antigens. A P-value <0.05 was considered statistically significant. Based on this, the cut-off value for α-PLG positivity was at the 97.5^th^ percentile (mean + 2 standard deviation) of the healthy controls. All statistical calculations were performed using SPSS (v25.0; IBM Corp, Armonk, NY).

## Results

### Setting up the anti-plasminogen assay

We started with glu-PLG as antigen, and set up an ELISA using different serum dilutions (1:50, 1:100, 1:200, 1:400) and purified IgG from the samples. Results showed unspecific binding and therefore IgG was removed from the antigen samples by running the PLG antigens through a Protein G affinity column. Most of the background binding disappeared and protein G purified PLG was used in all experiments after this.

Unspecific background binding was still present after purification of the antigen and, therefore, different set-ups were tested to obtain differentiation between a strong positive control and negative controls (i.e. one healthy control and a sample with only a saline buffer). Different combinations of coating buffers, blocking agents and diluents were tested. Optimal results distinguishing positive from negative controls were obtained with the combination carbonate buffer for coating and blocking with 1% BSA. Taking this set-up as baseline, we took further steps to optimize the assay. For the anti-human IgG conjugate we compared Sigma goat anti-human IgG HRP, Sigma goat anti-human IgG AP, and Dako rabbit anti-human IgG HRP. The Sigma goat anti-human IgG AP differentiated best between the positive and negative controls.

### Specificity for glu-plasminogen

**Fig 1:**
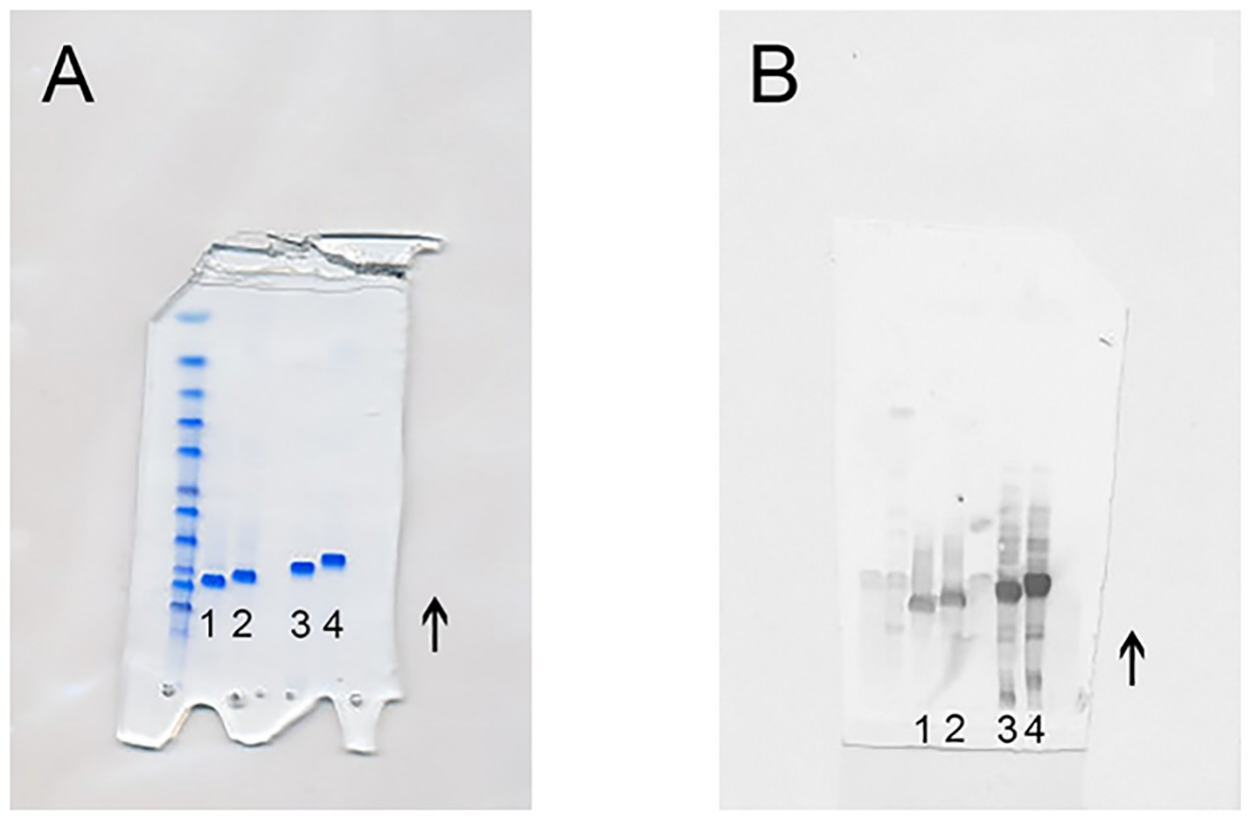
SDS-PAGE and Western blot with rabbit anti-plasminogen antibodies. (A) SDS-PAGE showed the presence of four 4 proteins. (B) Western blot with rabbit anti-plasminogen antibodies showing strong binding for the unreduced plasminogen variants and weak binding for the reduced plasminogen variants. 1, reduced glutamic acid plasminogen; 2, reduced lysine-plasminogen; 3, unreduced glutamic acid plasminogen (88 kDa); 4, unreduced lysine-plasminogen (83 kDa). The arrows show the direction of movement of the proteins.

Minimal inhibition was obtained in a set-up where human PLG was added as an inhibitor to an ELISA with glu-PLG as the epitope, in contrast to a control condition in which rabbit α-PLG IgG was inhibited by soluble human PLG. Therefore, SDS-PAGE and Western blot were performed to test to which protein structure the α-PLG bind best. SDS-PAGE showed the presence of four proteins: unreduced glu-PLG, unreduced lys-PLG, reduced glu-PLG, reduced lys-PLG. No other protein was detected. When Western blot was performed with rabbit α-PLG, it showed strong binding for the unreduced PLG variants and weak binding for the reduced PLG variants (Fig 1). This indicates the importance of epitope conformation and this probably is true also for humans *in vivo*, explaining why different forms of the proteins react with different patient sera.

### Lys-plasminogen as antigen coating

Lys-PLG had a more saturated titration curve than glu-PLG with maximal saturation at lys-PLG 3 μg/ml. An assay with purified glu-PLG coating 2,5 μg/ml, 1/100 diluted samples, was performed on 8 positive controls, 22 ANCA samples and 13 healthy controls. Of the 8 positive controls, in this assay only two were positive. When performing the same assay with purified lys-PLG coating 2,5 μg/ml, the same two positive controls were positive, but in addition, there were three positive controls which were weakly positive. In both assays, none of the ANCA samples showed α-PLG positivity.

**Fig 2:**
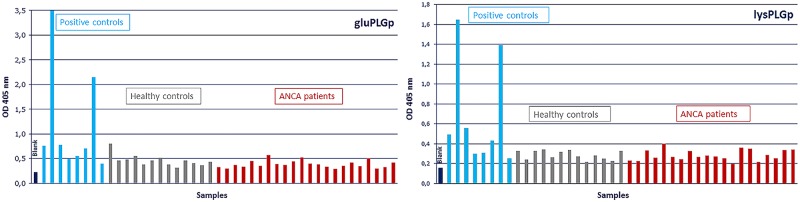
Spectrophotometrical results. (A) Spectrophotometrical results using purified glutamic acid plasminogen as an antigen in the assay. Optical density was measured with spectrometry using light of 405 nanometer for the detection of anti-plasminogen antibodies in the serum samples. (B) Spectrophotometrical results using purified lysine-plasminogen as an antigen in the assay. Optical density was measured with spectrometry using light of 405 nanometer for the detection of anti-plasminogen antibodies in the serum samples. Purified lysine-plasminogen showed more spectrophotometrical differentiation between positive samples and negative samples compared to purified glutamic acid plasminogen (Fig 2A). Abbreviations: gluPLGp, purified glutamic acid plasminogen; OD, optical density; nm, nanometer; lysPLGp, purified lysine-plasminogen.

Using purified lys-PLG showed more spectrophotometrical differentiation between positive samples and negative samples compared to purified glu-PLG. In the case of using glu-PLG as coating antigen, the difference was not statistically significant (P = 0.058). When using lys-PLG, the difference was statistically significant (P = 0.001). Therefore, the assay was further optimized using lys-PLG as the coating antigen (Fig 2). We performed several assays with PLG antigens from different suppliers, different coating concentrations, serum dilutions and time periods at room temperature and at 37°C. All this was done in order to reduce unspecific binding and increase specific binding.

### Testing the current assay

**Fig 3:**
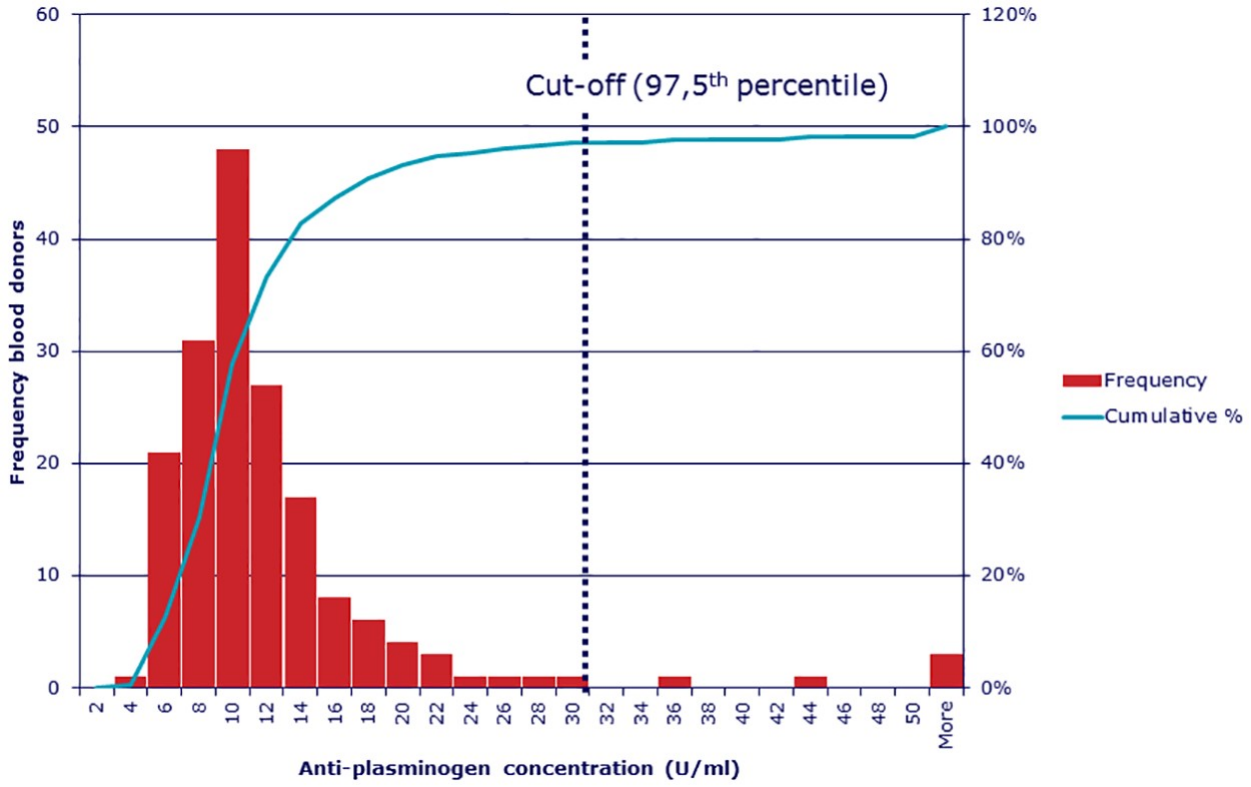
Anti-plasminogen antibody concentrations in serum samples from 175 healthy controls. The cut-off value for anti-plasminogen antibodies positivity was set to 31 U/ml and higher based on the 97.5^th^ percentile of the healthy controls.

With the current assay (Box 1), we tested a new cohort of samples. This cohort consisted of 75 ANCA positive patients (35 MPO-ANCA, 40 PR3-ANCA), 135 disease controls (anti- β2GP1 positive [n = 55], anti-CCP positive [n = 80]), 175 healthy controls, 11 positive controls (5 MPO-ANCA, 5 PR3-ANCA and 1 ANCA negative) and 1 negative control (healthy control who was negative in all previous tests). The cut-off value for α-PLG positivity was 31 U/ml and higher based on the 97.5^th^ percentile of 175 healthy controls (Fig 3). Table 1 shows the results.

**Table 1:** Overview of anti-plasminogen antibody positive patients using our optimized assay.

Samples	α-PLG positive patients	α-PLG negative patients	Total patients	% α-PLG positive patients
MPO-ANCA	5	30	35	14.3
PR3-ANCA	0	40	40	0.0
Anti-β2GP1	11	44	55	20.0
Anti-CCP	10	70	80	12.5
Positive controls	7	4	11	63.6
Negative control	0	1	1	0.0

Abbreviations: α-PLG, anti-plasminogen antibodies; ANCA, anti-neutrophil cytoplasmic antibody; MPO, myeloperoxidase; PR3, proteinase-3; anti-β2GP1, anti-beta-2 glycoprotein 1; anti-CCP, anti-cyclic citrullinated peptides.

## Discussion

We developed an optimized assay for the detection of α-PLG, focusing on its usefulness in studies on AAV. We tested different assay set-ups with for example different types of PLG antigens and coating buffers. Importantly, we found that purified lys-PLG showed better spectrophotometrical differentiation between positive and negative samples than glu-PLG when used as a coating antigen (Fig 2).

Using our assay, we found that 14.3% of MPO-ANCA patients tested had α-PLG, whereas all our PR3-ANCA patients tested negative. However, α-PLG were detected in PR3-ANCA samples from our positive controls. Our results show discrepancies with previous studies. Bautz *et al*. described the presence of α-PLG in 22% (16/72) of patients with PR3-AAV and in 6% (2/34) of patients with MPO-AAV. In the PR3-AAV group, this proportion was significantly higher compared to healthy and disease controls. There was no difference between MPO-AAV and the controls.[1] Berden *et al*. found α-PLG in approximately 25% of patients with PR3-ANCA and with MPO-AAV. Both were significantly higher than in healthy and disease controls.[2] Hao *et al*. detected α-PLG in 42.8% (3/7) of PR3-AAV patients and in 16.4% (16/97) of MPO-AAV patients. The proportion of AAV-patients with α-PLG was significantly higher than in healthy controls.[3] Although our positive control samples came from the studies of Bautz *et al*. and Berden *et al*.,[1,2] the discrepancy can be explained by the following: in the present study we have combined important technical findings/methods from the previous studies, which have led to the optimized α-PLG assay here presented. A known challenge in the α-PLG assay is the chance for false positive results. We optimized each step in the assay by testing different alternatives taking into account the assays used in the previous studies on α-PLG in AAV. Therefore, our assay is slightly different from the assays used previously, which could partly explain the discrepancy. The ultimate experimental set-up investigating what could have caused the discrepancies of the studies thus far conducted would entail that the optimized assay developed by us would be used on selected patients from the previous studies and in parallel with the assays used for these studies. Unfortunately, this set-up lay beyond the scope of the current experimental design.

In our assay we used purified PLG proteins as coating antigens. The PLG delivered by vendors is purified from blood, which means it can contain trace amounts of IgG. This will cause unspecific binding in the assay giving high background signals and therefore we decided to purify the PLG delivered by the vendor. This could also be a possible explanation for the discrepancies of our study with previous studies. Only Hao *et al*. described the purification of the PLG coating protein in their assay, but they did not mention whether they purified it themselves or whether it was assumed to be purified.[3] This could be the most critical confounder that other studies encountered, if a protein was purchased and assumed to be purified, but in reality contained considerable amounts of contaminating IgG.

Originally, α-PLG were described in view of anti-complementary PR3 antibodies which were suggested to develop within an idiotypic antibody response.[1,7] This scenario assumed a typical combination of PR3-ANCA and α-PLG. With the repetitive finding of MPO-AAV patients having α-PLG in the absence of PR3-ANCA antibodies, this hypothesis became less likely than previously thought.

In previous studies, Bautz *et al*. and Hao *et al*. included diagnostic sera from patients with active disease at baseline, which is equal to the time of diagnosis.[1,3] In the study by Berden *et al*., a few patients were included who were in clinical remission at baseline.[2] These studies showed data suggesting a transient nature of α-PLG. Be it in only a few cases (n = 3), Berden *et al*. did show disappearance of the α-PLG in two patients after treatment; however, there was also one patient in remission who remained positive for α-PLG.[2] In the study by Bautz *et al*., nine PR3-ANCA positive patients with thrombotic events during follow-up were reported, and five of them were positive for α-PLG in the presence of active disease, while the others were in remission and were negative for α-PLG at the time of the thrombotic event.[1] Hao *et al*. described 48 patients with sequential samples of whom 7 were positive for α-PLG during active disease; at remission during follow-up only one of the patients remained positive. In fact, Hao *et al*. noted that there was a better association between the levels of α-PLG and disease activity compared to ANCA levels and disease activity.[3] Therefore, these results suggest that α-PLG may be transient in nature. However, no firm conclusions can be drawn because of limited available data. The exact biology behind the occurrence and disappearance of these antibodies is not known and needs further study. In our study, samples were not selected with respect to disease state, since this was beyond the scope of our study.

The various studies on α-PLG in AAV had differences in their assays, which could have been responsible for the discrepancies in the results.[1–3] Different concentrations of coating antigen and different conjugated secondary antibodies were used to detect antibody-antigen complexes. In order to prevent false positive results, we optimized each step in the assay and used purified PLG as coating antigen to prevent background and unspecific binding. One assay used sera as samples while others used purified IgG with different definitions for positivity. In the previous studies a 97.5th percentile (mean + 2 standard deviation) threshold of healthy controls was used as a cut-off point for α-PLG positivity. Hao *et al*. tested the samples one time.[3] Bautz *et al*. analyzed the highest value measured, not mentioning how often a sample was tested.[1] Berden *et al*. tested all samples 6 times and considered a patient α-PLG positive when in >50% of the occasions the assay was positive.[2] In our study samples were tested one time for α-PLG positivity. In addition, there was little information in these studies regarding the PLG antigen used for coating. Only Hao *et al*. specified their antigen further describing that it was human PLG supplied by Abcam (Cambridge, UK). The product information describes that this is a full length natural human PLG protein, so we assume that this probably is glu-PLG.[3] We demonstrated that using lys-PLG as a coating antigen showed better differentiation between negative and positive controls.

An important discussion point regarding α-PLG remains its epitope-specificity. Also in the field of ANCA-specificity this still is an ongoing discussion. It is thought that varying epitope-specificity of ANCA will influence their physiological effects and their potential for pathogenicity. Currently described factors that influence the detection of ANCA and the assessment of their pathogenicity are epitope specificity, masking of (pathogenic) epitopes, modified antigens and technical limitations of current assays.[8–10] Several epitopes relevant for PR3-ANCA AAV and MPO-ANCA AAV are described in the literature. These are linear and conformational epitopes. Especially, the conformational epitopes form a major problem for diagnostic purposes and pathogenic studies.[8–10] Similar issues will form a challenge in future research for α-PLG. Of the previous studies on α-PLG in AAV, only Bautz *et al*. described the target epitope of the detected α-PLG, which was also found on complementary PR3 as discussed previously.[1] Glu-PLG and lys-PLG are biologically correlated; removing the PAp domain—which consists of several amino acids—changes glu-PLG into lys-PLG.[11] In the activation process, glu-PLG and lys-PLG are both open conformations, which are distinct from each other.[12] Open conformation glu-PLG and open conformation lys-PLG can both be converted to plasmin by tPA or uPA. Glu-PLG can first be converted into lys-PLG and then into plasmin or glu-PLG can directly be converted into plasmin. Unfortunately, little is known about the exact mechanism of conformational changes of PLG in its different states.[13] Reactivity to each conformational state may be important in different ways biologically and must be further investigated for their pathogenic characteristics and potential diagnostic purposes.

In the present study we have combined important technical findings/methods from previous studies which have led to the optimized α-PLG assay here presented. Until now, there were many uncertainties and differences in the setup and results of hitherto used assays. Our optimized assay can be used for future research purposes and will aid in uniform reporting on α-PLG, opening the way to further explore their relevance in AAV.
